# Endoparasite Prevalence in the Mountain Bongo (*Tragelaphus eurycerus* spp. *isaaci*) at Mount Kenya Wildlife Conservancy

**DOI:** 10.1002/ece3.73347

**Published:** 2026-03-31

**Authors:** Samuel Njuki Mahiga, Evans M. Mwangi, Robert Aruho, Joyce Omari, Paul W. Webala

**Affiliations:** ^1^ Department of Biology University of Nairobi Nairobi Kenya; ^2^ Mount Kenya Wildlife Conservancy Nanyuki Kenya; ^3^ Maasai Mara University Narok Kenya

**Keywords:** Coccidia, endoparasite, mountain bongo, Strongylids

## Abstract

Parasite–host interactions influence the success of wildlife reintroductions. The mountain bongo (*Tragelaphus eurycerus isaaci*) is a critically endangered antelope found only in Kenya's montane forests, with fewer than 100 remaining in the wild. It is, therefore, imperative to re‐establish a viable, healthy, and self‐sustaining population in its native habitat. We analyzed the fecal samples from a re‐established population over a two‐year period, identifying Coccidia and Strongylids as the predominant gastrointestinal parasites. Infestation levels for both were higher during the wet season than the dry season and higher during the calf age followed by sub‐adult and least at adult stage. The prevalence of both Coccidia and Strongylids differed significantly by age group, but only Coccidia differed significantly by season. Understanding variation in parasite load can assist in planning for priority interventions such as boosting nutrition, administering medication, or employing other methods to counter high‐risk season and age group infections. Our study also contributed to understanding differences in survival rates and shed light on how these are related to parasite load. Results emphasize the need to conduct more studies on the bongo, its habitat, and drivers of parasite load species sharing the ecosystem.

## Graphical abstract

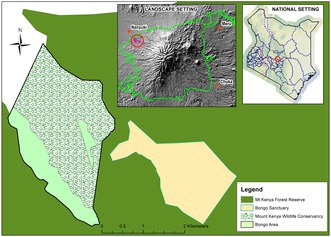

## 1 Introduction

There is increasing interest in understanding how parasite–host interactions influence ecosystem functionality and biodiversity (Giari et al. 2020; Hatcher et al. 2012; Preston and Johnson 2010). The introduction of wildlife into new environments may trigger changes that affect the dynamics of both (Calero‐Bernal and García‐Bocanegra 2023). Parasites impact wildlife health and fitness (Omeragić et al. 2024) influence host population demographics (Staples et al. 2022) and shape community structure (Dobson et al. 2008; Valdovinos et al. 2023). They influence food webs and, consequently, ecosystem stability (Hatcher et al. 2012; Preston and Johnson 2010). Infection levels are typically low and generally asymptomatic (Mugendi 2013), but anthropogenic activities may alter transmission rates, host range, and virulence (Daszak et al. 2000). This poses an extinction risk (Chakuya et al. 2016; Obanda et al. 2011). Parasites and their hosts must therefore maintain a natural balance, although they may cause death and species decline (Mugendi 2013). The risk posed to endangered species is frequently disregarded until a serious issue arises (Muoria et al. 2005).

Reintroduction into the wild using captive bred individuals has become a well‐established conservation strategy (Moreno Mañas et al. 2019). Poor planning and oversight (diseases, weather, water, social aspects) have led to failure in many attempts to re‐establish viable populations (Fischer and Lindenmayer 2000). Factors that influence the success of reintroduction include parasites, diseases, predators, food, competition, and weather. These may have an impact on a population's ability to grow, survive and reproduce (Germano et al. 2014; Taylor et al. 2017). The costs of parasitism have wide‐ranging effects on host growth, reproduction, susceptibility and mortality (Bethony et al. 2006; Fischer and Lindenmayer 2000; Hotez et al. 2008).

The mountain bongo has a declining isolated population of less than 100 individuals left in the wild (WRTI 2021; KWS 2019; IUCN 2017). In contrast, around 500 persist in captivity around the world, including approximately 52 at a captive breeding facility within Kenya (Sheppard et al. 2022). The captive population in Kenya was acquired from the American Zoo Association (AZA) facilities in the USA and repatriated to the MKWC in 2004. The conservancy received 14 females and four males with the aim of re‐establishing a viable and self‐sustaining population in the native habitat (CBSG 2010; KWS 2019).

Unfortunately, 75% of the repatriated animals died of Theileria spp. (KWS 2019) and other parasite‐related infections (CBSG 2010; Kock et al. 2007), with infant mortality claiming 50% of all births during the same period (King'ori et al. 2025). In 2019, MKWC lost 38 bongos to parasite‐related complications, with diseases and parasites being among the major impediments to recovery. This study sought to document gastrointestinal parasite prevalence in mountain bongo.

## 2 Materials and Methods

### 2.1 Study Area

**FIGURE 1:**
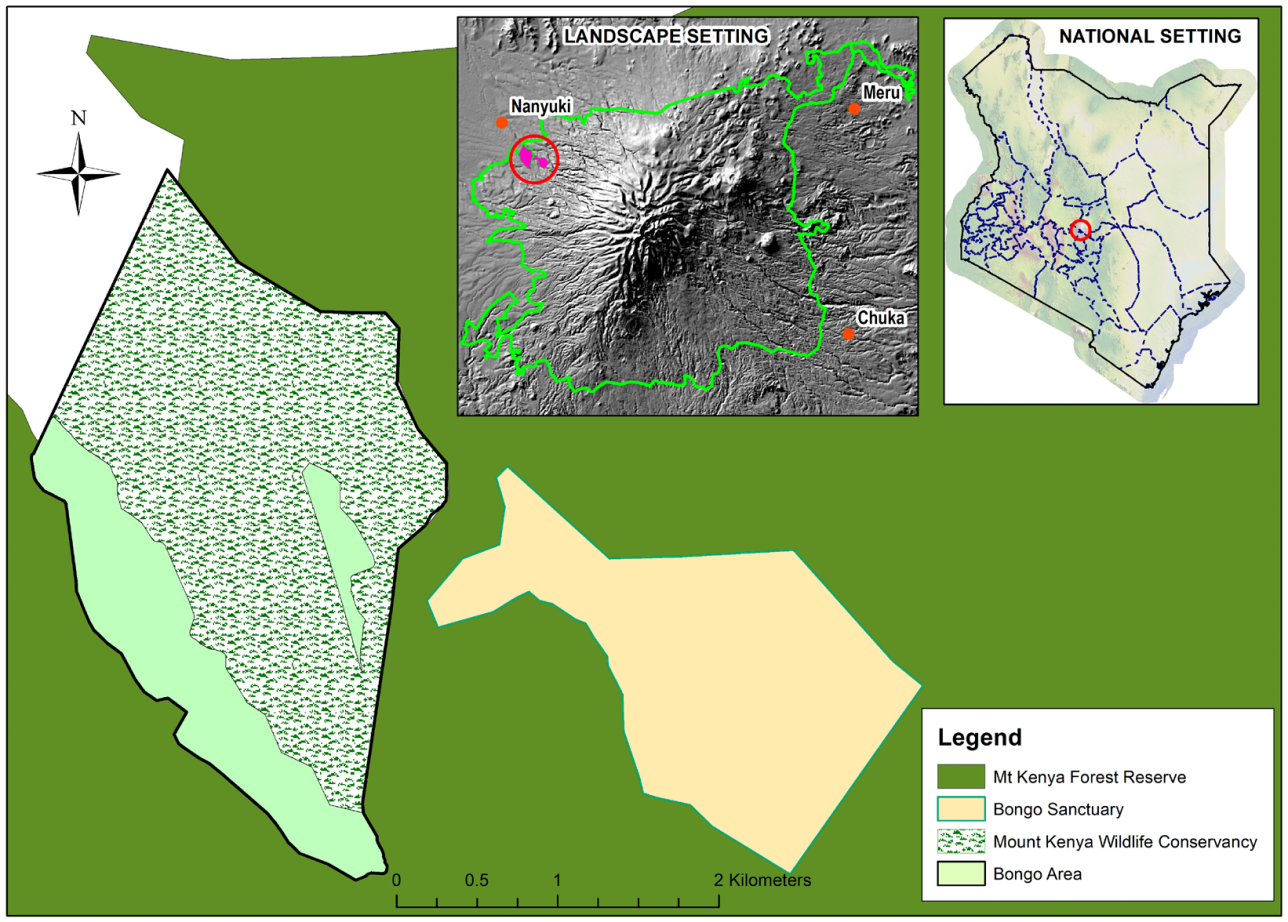
Study area map.

The study was conducted at MKWC a UNESCO world heritage site (King'ori et al. 2025), and the adjacent Mawingu Mountain Bongo Sanctuary (MMBS) which cover 1250 and 776 acres, respectively. The area is located on the slopes of Mt. Kenya centered around latitude 0°2′40.00″ S and longitude 37°8′26.27″ E, and lying at an altitude of 2387 m above sea level (Figure 1).

**FIGURE 2:**
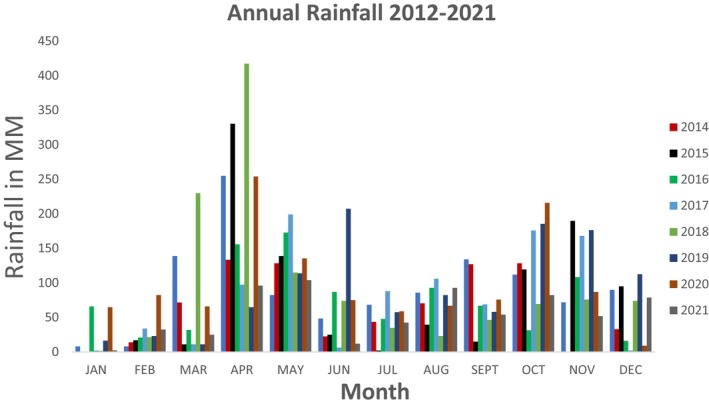
Rainfall trends 2012–2022 (*Source:* William Holden Wildlife Foundation).

Annual rainfall shows a bimodal pattern (King'ori et al. 2025), with two dry seasons and two wet seasons (Figure 2) where the long rains are experienced between March and May and short rains between October and November (Fundi 2013). The area is important for overall biodiversity conservation, is a main water tower, and designated as an Important Bird Area and world heritage site.

### 2.2 Fecal Sample Collection and Parasitology

Fecal samples (*n* = 1649) were collected over 2 years during focal individual observations from 89 individuals during wet and dry seasons. Individuals were randomly selected and whenever an individual was observed defecating, fresh dung that was not in direct contact with soil was opportunistically collected from the ground within a few minutes. The samples were homogenized, immediately placed in a cooler box with ice packs and transported to MKWC laboratory for processing within a maximum period of 2 h.

Two grams of the homogenized fecal sample were mixed with 28 mL of Epsom salt (MgSO_4_) solution (Zajac et al. 2021) and the slurry was sieved through a tea strainer. The filtrate was transferred to a 15 mL centrifuge tube and after agitation, an aliquot was taken from the tube and pipetted into McMaster slide chambers. The slides were set aside for 5 min before being examined under a light microscope to allow parasite eggs to float to the surface. All eggs inside the grid areas were counted using the 10× objective. We examined four slide preparations from each sediment sample using the McMaster dilution egg counting technique (Preston 2024; Zajac et al. 2021). All collected fecal samples were analyzed using Labo American Inc. USA Microscope.

### 2.3 Data Analysis

The generalized linear mixed‐effects models (GLMMs) were fitted using the nlme package (Pinheiro and Bates 2000) in R, to investigate how the abundance of *Coccidia* and *Strongylida* ectoparasites varied with host age and sex groups, as well as between dry and wet seasons. To account for repeated observations of the same individuals, we included individual identity as a random effect in all models.

Because both response variables (gastrointestinal parasite abundances) consisted of count data and exhibited a high frequency of zero values, we assumed a negative binomial error distribution for model fitting. For each response variable, a candidate set of four competing models was constructed representing different combinations of the explanatory variables. Model selection was based on the small‐sample–corrected Akaike's Information Criterion (AICc), with the lowest AICc value being considered the most parsimonious and best‐supported within each response category.

## 3 Results

Two taxa of parasites (*Coccidia* and *Strongylida*) were identified across the samples. Analyses of gastrointestinal parasite burdens revealed that host age is a strong and consistent predictor of infection intensity for both coccidia and Strongylids, whereas seasonal effects and sex differences varied between parasite taxa.

### 3.1 Influence of Age, Season and Sex on *Coccidia* Egg Count in Mountain Bongo

**FIGURE 3:**
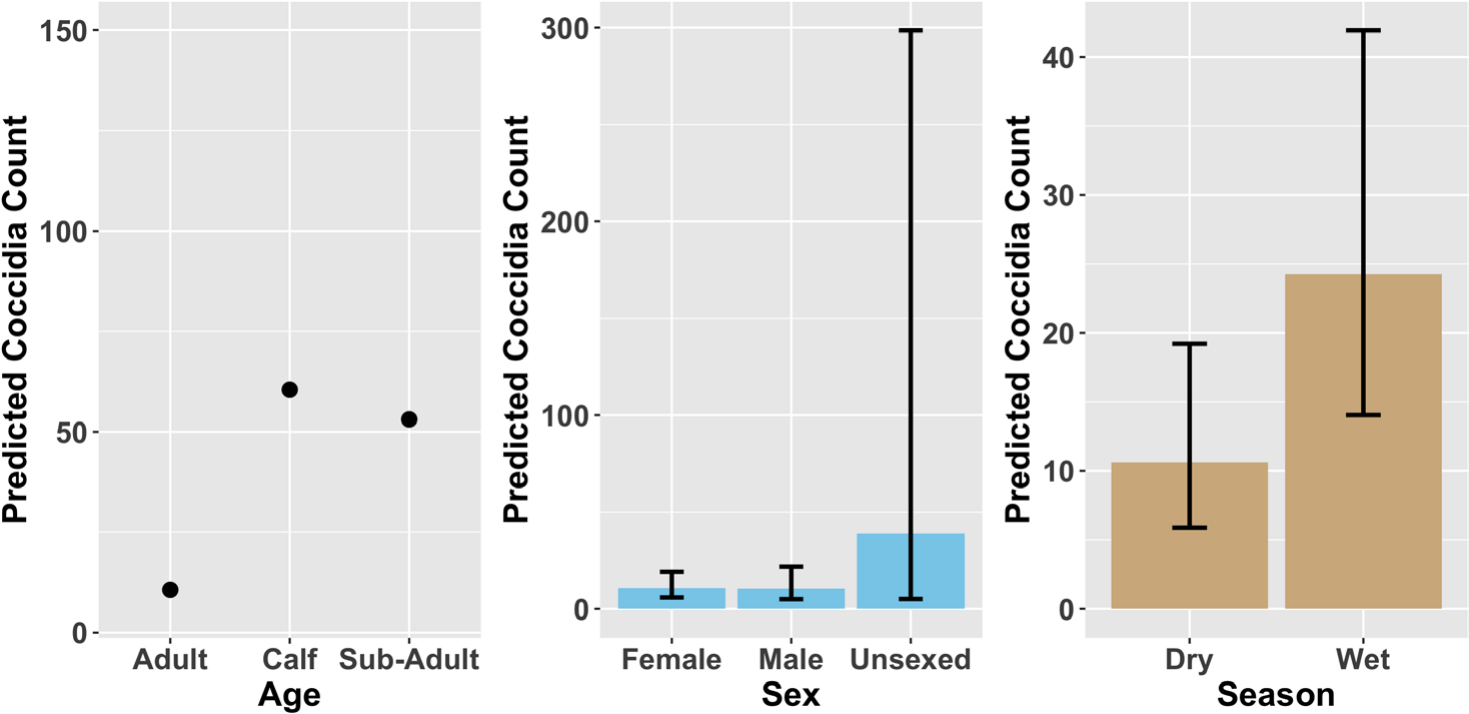
Influence of age, sex, and season on Coccidia egg count in mountain bongo.

Coccidia counts were significantly associated with age and season in the negative binomial GLMM (Figure 3). Compared with the other age group, calves exhibited substantially higher coccidia counts (*z* = 4.08, *p* < 0.001), as did sub‐adults (*z* = 3.35, *p* < 0.001). Coccidia counts were also significantly higher during the wet season compared to the dry season (*z* = 2.40, *p* = 0.016). In contrast, sex had no detectable effect on coccidia counts (male: *p* = 0.97; unsexed: *p* = 0.20). The random intercept for individual identity accounted for additional variation in baseline infection levels (SD = 0.17), indicating moderate among‐individual heterogeneity.

### 3.2 Influence of Age, Season and Sex on Strongylids Egg Count in Mountain Bongo

**FIGURE 4:**
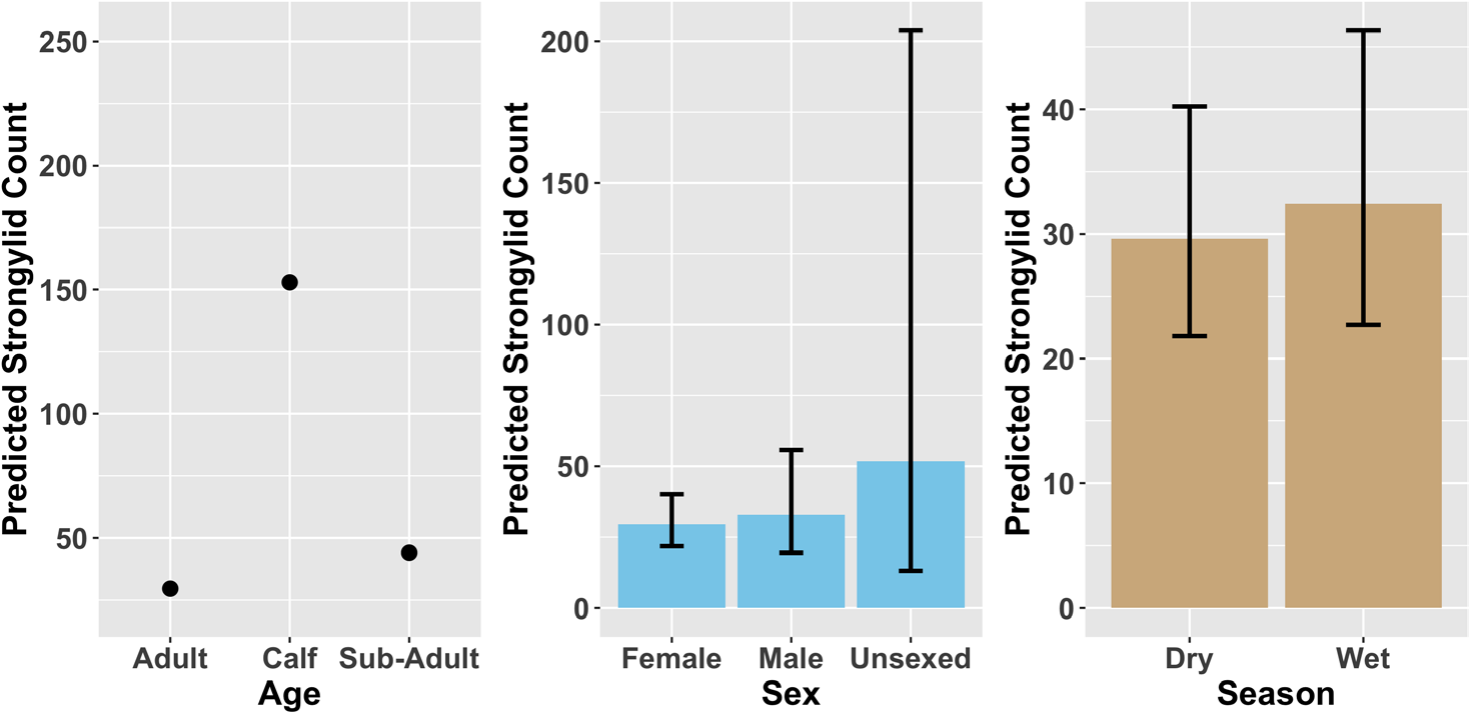
Influence of age, sex and season on Strongylid egg count in mountain bongo.

Strongylid counts were primarily influenced by age, with calves showing significantly higher parasite counts compared with the other age group (*z* = 6.01, *p* < 0.001) (Figure 4). In contrast, sub‐adults did not differ significantly from the reference group (*z* = 1.27, *p* = 0.203). Neither season nor sex had a significant effect on Strongylid counts (wet season: *z* = 0.44, *p* = 0.662; males: *p* = 0.667; unsexed: *p* = 0.413). The random intercept for individual identity explained negligible variation in baseline Strongylid infection levels (SD < 0.001), indicating minimal among‐individual heterogeneity after accounting for fixed effects.

## 4 Discussion

Understanding gastrointestinal parasite dynamics in endangered ungulates is critical for both disease ecology and conservation management, particularly for species such as the mountain bongo which persist in small, vulnerable populations (Albery 2022; Heckley and Becker 2024; King'ori et al. 2025). This study revealed two gastrointestinal parasites—coccidia (phylum Apicomplexa), a diverse group of intracellular protozoa widely spread in vertebrates (Duszynski et al. 2007; Kyzy 2010; Tenter et al. 2002) and Strongylids, which are nematodes with a complex life cycle that involves multiple stages of development within the host and on the pasture (Hamad et al. 2024). The presence of *Strongyloides* spp. is particularly concerning (Obanda et al. 2019) as these parasites directly damage the intestinal epithelium and cause anemia, weight loss, diarrhea, and mortality, especially in young or immunocompromised hosts (Obanda et al. 2011; Omeragić et al. 2024; Preston 2024). Given the compounded threats posed by disease, genetic inbreeding, and habitat fragmentation, targeted research initiatives and specialized veterinary interventions remain imperative to ensure the long‐term viability of this critically endangered species (King'ori et al. 2025).

Across both parasite taxa, host age consistently emerged as the most robust predictor of infection intensity, a trend that aligns with well‐documented age‐related patterns in wild mammal–parasite systems (Clerc et al. 2019; Parker et al. 2020; Wilson et al. 2002). Bongo calves exhibiting significantly higher coccidia and Strongylid counts than older individuals this was consistent with evidence that juvenile hosts experience elevated infection risk due to immunological immaturity and limited prior exposure (Clerc et al. 2019). This observation was consistent with previous studies that have documented higher egg count in calves than subadults and adults (Del Río et al. 2025; Herskind et al. 2023; Wood et al. 2013). This could also be related to management practices, such as supplemental feeding, which may cause an increase in the concentration of the animals around feeding areas (Herskind et al. 2023; Kołodziej‐Sobocińska et al. 2016). Early‐life parasite exposure is increasingly recognized as a key determinant of lifetime infection trajectories and fitness outcomes in wild ungulates (Froy et al. 2019). Such findings highlight the importance of targeted parasite control programs for calves and subadults to minimize health risks and support population recovery (Staples et al. 2022). Wildlife managers should therefore implement age‐specific parasite management strategies (Lafferty et al. 2006; Omeragić et al. 2024).

Although calves showed elevated infection for both parasite taxa, age‐related patterns diverged beyond early life, with coccidia remaining high in sub‐adults while Strongylid burdens declined. This reflected differences in parasite biology and host immune responses (Hawlena et al. 2006; Tomczuk et al. 2015), a phenomenon crucial for understanding host–parasite interactions and developing effective control and management strategies (Kimeli et al. 2020).

Coccidia typically induce partial, exposure‐dependent immunity, allowing continued oocyst shedding even after immune priming, particularly in environments with high contamination pressure (Bangoura and Bardsley 2020). In contrast, Strongylid nematodes, which are extracellular parasites, exhibit a different age‐related infection pattern, being common and capable of causing significant health issues, particularly in young animals (Claerebout et al. 2005). Initial Strongylid infections are often observed in young calves, with prevalence and intensity varying according to environmental factors, management practices, and host immunity (Ahn et al. 2024; Kimeli et al. 2020). As the host matures, however, there is a general decline in Strongylid burdens (Ahn et al. 2024; Hämäläinen et al. 2015; Hawlena et al. 2006). This is primarily driven by the development of a robust adaptive immune response in older animals (Claerebout et al. 2005).

Season significantly influenced coccidia infection intensity, with a pattern consistent with recent studies demonstrating strong environmental control of protozoan transmission (Jansen et al. 2020). Increased moisture enhances oocyst survival and sporulation, leading to greater environmental contamination and ingestion risk during foraging. (Mugendi 2013). In forest‐dwelling ungulates such as the mountain bongo, persistently humid microhabitats can intensify wet‐season transmission dynamics by promoting the survival and infectivity of environmentally resistant parasite stages. In contrast, Strongylid infections showed no detectable seasonal variation, suggesting that host immunity or stable exposure may dampen environmentally driven fluctuations.

Parasite load is paramount in wildlife conservation and reintroduction programs, as it determines the future health and survival of both introduced populations and those already in the habitat (Rush et al. 2021). As this study demonstrated, understanding this provides vital insights into the challenges facing endangered species and emphasizes the importance of integrating parasite load assessments into conservation strategies, particularly for species being reintroduced into their natural habitats. Adult parasites damage the intestinal lining, causing anemia, weight loss, diarrhea, colic, and, in severe cases, death, particularly in young or debilitated animals (Johnson et al. 2021; Robi et al. 2023; Thumbi et al. 2014). As such, targeted parasite control programs for calves and subadults could minimize health risks and support population recovery (Robi et al. 2023; Staples et al. 2022).

High parasite burdens are known to reduce reproductive success, compromise host health, and elevate mortality in wildlife populations. Juvenile cohorts often exhibit disproportionately higher prevalence compared to adults and sub‐adults (Altizer et al. 2003; Ezenwa 2004). In the case of the MKWC, such parasitic pressures may be a key driver of the slow demographic recovery observed, thereby constraining breeding success and limiting rewilding initiatives (King'ori et al. 2025).

This study suggested that parasite load should be considered in future reintroduction. Targeted health assessments and management strategies should be carried out based on sex, age and seasons, to help tailor mitigation and address specific vulnerabilities. It also demonstrated the necessity of monitoring the previously reintroduced populations (Fundi et al. 2022; Sheppard et al. 2022). Regular fecal examinations can inform timely interventions, such as deworming or habitat modifications. These could help maintain the health and stability of the population after release, contributing to the overall success of the conservation program.

Understanding gastrointestinal parasite dynamics in the mountain bongo is primarily determined by host age and parasite life history, and secondarily influenced by seasonality (King'ori et al. 2025). This underscores the necessity of age‐ and parasite‐specific approaches in wildlife disease ecology and conservation (King'ori et al. 2025). Targeted monitoring of young individuals during high‐risk seasons, coupled with tailored health assessments and management strategies, is vital for safeguarding the future of this critically endangered species (King'ori et al. 2025; Sandri et al. 2023).

## 5 Conclusion

We recommend that management teams maintain regular monitoring of parasite prevalence and infection intensity within the bongo population, with particular focus on calves and subadults, which face higher susceptibility to infection. Furthermore, implementing a targeted and evidence‐based endoparasite control program will reduce infection pressure while limiting the development of anthelmintic resistance. By integrating sustained surveillance with strategic parasite control, conservation managers can strengthen reintroduction success and support the long‐term recovery of this endangered species.

## Author Contributions

**Samuel Njuki Mahiga:** conceptualization (lead), data curation (lead), formal analysis (lead), funding acquisition (lead), investigation (lead), methodology (lead), project administration (lead), resources (lead), software (lead), supervision (lead), validation (lead), visualization (lead), writing – original draft (lead), writing – review and editing (lead). **Evans M. Mwangi:** conceptualization (lead), data curation (lead), formal analysis (supporting), funding acquisition (supporting), investigation (supporting), methodology (supporting), project administration (supporting), supervision (lead), writing – review and editing (supporting). **Robert Aruho:** conceptualization (supporting), data curation (supporting), funding acquisition (supporting), investigation (supporting), writing – review and editing (supporting). **Joyce Omari:** conceptualization (supporting), data curation (supporting), formal analysis (supporting), funding acquisition (supporting), investigation (supporting), methodology (supporting), validation (supporting), visualization (supporting), writing – original draft (supporting), writing – review and editing (supporting). **Paul W. Webala:** conceptualization (supporting), data curation (supporting), formal analysis (supporting), funding acquisition (supporting), investigation (supporting), methodology (supporting), project administration (supporting), resources (supporting), software (supporting), supervision (lead), validation (supporting), visualization (supporting), writing – original draft (lead), writing – review and editing (lead).

## Funding

This study was supported by Wildlife Conservation Network and Rufford Foundation (38463‐1).

## Conflicts of Interest

The authors declare no conflicts of interest.

## Supporting information


**Data S1:** ece373347‐sup‐0001‐DataS1.R.

## Data Availability

We have provided the data as Supporting Information.
